# The Impact of a Manufacturing Process on the Stability of Microcrystalline Long-Acting Injections: A Case Study on Aripiprazole Monohydrate

**DOI:** 10.3390/pharmaceutics17060735

**Published:** 2025-06-03

**Authors:** Tomasz Pietrzak, Ziemowit Szendzielorz, Joanna Borychowska, Tomasz Ratajczak, Marcin Kubisiak

**Affiliations:** Research and Development Department, Pharmaceutical Works Polpharma S.A., Pelplińska 19, 83-200 Starogard Gdański, Poland; ziemowit.szendzielorz@polpharma.com (Z.S.); joanna.borychowska@polpharma.com (J.B.); tomasz.ratajczak@polpharma.com (T.R.)

**Keywords:** long-acting injection (LAI), stability, bead milling, sterilization, high-shear homogenization

## Abstract

**Background/Objectives:** Long-acting injections (LAIs) are innovative drug delivery systems that improve patient compliance by maintaining therapeutic drug levels over extended periods. Micro- and nanosuspensions are commonly used in LAIs to enhance bioavailability, but their thermodynamic instability poses challenges, including particle aggregation and growth. This study aimed to evaluate the impact of two helping processes—vehicle thermal treatment and high-shear homogenization—on the stability and manufacturing efficiency of aripiprazole monohydrate (AM) suspensions. **Methods:** AM suspensions containing sodium carboxymethyl cellulose (CMCNa), mannitol and disodium phosphate in water for injections (WFIs) were prepared using a combination of thermal treatment of the vehicle solution, high-shear homogenization and bead milling. Four manufacturing variants were tested to assess the influence of these processes on particle size distribution (PSD), viscosity and stability during a 3-month accelerated stability study. Molecular weight changes in CMCNa from thermal treatment were analyzed using size exclusion chromatography with multiangle scattering (SEC-MALS), and PSD was measured using laser diffraction. **Results:** Thermal treatment of the vehicle solution had minimal impact on CMCNa molecular weight, preserving its functionality. High-shear homogenization during bead milling significantly reduced particle aggregation, resulting in improved PSD and reduced viscosity. Synergistic effects of the two helping processes used in one manufacturing process were observed, which led to superior stability and minimal changes in PSD and viscosity during storage. Batches without the helping processes exhibited increased particle size and viscosity over time, indicating reduced suspension stability. **Conclusions:** Incorporating vehicle thermal treatment and high-shear homogenization during bead milling enhances the stability and manufacturing efficiency of AM suspensions. These findings underscore the importance of optimizing laboratory-scale processes to ensure the quality and safety of pharmaceutical suspensions.

## 1. Introduction

Long-acting injections (LAIs) represent a groundbreaking approach to drug delivery, particularly within the fields of psychiatry [1,2,3], endocrinology [4,5] and infectious diseases [6,7,8]. These innovative formulations are designed to maintain therapeutic drug levels in the body over extended periods, ranging from weeks to months, thus enhancing patient compliance and improving clinical outcomes [9]. A variety of formulation techniques are used for the manufacturing of LAIs including micro- and nanocrystalline drug suspension [10,11], oil-based solutions [12] and polymer-based systems with microspheres and in situ-forming (ISF) polymer implants [13,14]. The diversity of these approaches is crucial to achieve a stable drug formulation with precise pharmacokinetic profiles tailored to specific therapeutic needs [9,15,16].

A vast number of LAI marketed drugs are in the form of micro- and nanosuspensions, which enhance their bioavailability and dissolution rates [11,17]. In turn, these formulations are thermodynamically unstable, and their main drawback is the tendency to increase in particle size with time due to flocculation [18], particle growth by Ostwald ripening [19,20] or agglomeration [21,22]. Therefore, once the desired particle size is achieved, the suspension must be stabilized to prevent aggregation [22]. The long-term stability of micro- and nanosuspensions is a multifaceted challenge that requires a comprehensive understanding of formulation chemistry and manufacturing process parameters. Most studies in the former area focus on optimizing the concentration of stabilizers and surfactants, adjusting pH and controlling ionic strength to provide a stable dispersion [22,23,24]. In the case of drug substances with limited stability in aqueous solutions, the freeze-drying process can also be used to maintain the desired particle distribution after reconstitution [25]. However, our knowledge on the impact of the manufacturing process of micro/nanosuspensions on suspension stability is rather limited, and there are only a few articles that address this issue [26,27,28].

Two primary methodologies employed for micro- and nanosuspension preparation are the “top-down” and “bottom-up” approaches. The “bottom-up” approach involves assembling nanoscale particles from molecular or atomic components [11]. This method is typically based on precipitation or chemical synthesis, where the drug is dissolved and then precipitated to form nanoparticles. In turn, the key aim of the “top-down” methodology is the reduction in the particle size of the API to micro- or nanoscale [29]. This can be achieved through various techniques, with wet bead milling [30] and high-pressure homogenization [31,32] as noted examples. Both processes are commonly used in the pharmaceutical industry due to their scalability and repeatability, allowing for large-scale operations while maintaining the consistency and quality of a drug product. Wet milling involves using high-shear forces to break down drug particles by small milling beads [33]. The process is essentially time-consuming and requires significant energy input to achieve the desired particle size, which usually leads to high operational costs. Thus, ongoing research and development are crucial to increase the effectiveness of the milling process while reducing its duration to obtain a stable suspension. Recent advancements have focused on the optimization of the milling process to enhance efficiency and scalability. Key process parameters, including stirrer speed, bead loading and milling time, are usually tested to achieve the optimal particle size distribution [27,30,34,35,36]. Another approach involves the use of bead mixtures, combining small and large beads to balance collision frequency and energy utilization [30,34]. This strategy allows the smaller beads to efficiently reduce particle size during the later stages of milling, while the larger beads effectively break down coarse particles at the beginning of the process. Additionally, advanced computational tools, including mechanistic modeling and machine learning algorithms, are being employed to predict breakage kinetics and optimize process parameters. These tools enable precise control over the milling process, ensuring reproducibility and scalability for industrial applications [34]. However, to our knowledge, the impact of easy-to-implement industrial processes, e.g., high-shear homogenization or thermal treatment of a vehicle solution, on both the bead milling process and the stability of the resulting suspension has never been the subject of practical considerations.

The aim of our study is to investigate how the introduction of two supporting processes, i.e., thermal treatment of a vehicle solution and high-shear homogenization, influences the effectiveness of wet bead milling and the stability of the resulting LAI formulation. In this study, physicochemical parameters such as PSD and the viscosity of suspensions are compared after the milling process, both without and with the supporting processes. Additionally, molecular weight changes in CMCNa from thermal treatment of a vehicle solution are analyzed using SEC-MALS, and the stability results after 3 months of accelerated studies for the suspensions after lyophilization are presented. Taking into account that the thermodynamic instability of the drug substance during the bead milling process can lead to the formation of unstable polymorphs or amorphous forms, aripiprazole monohydrate (AM) has been chosen as a model drug, due to its relatively stable crystalline structure. AM is marketed by Otsuka Pharmaceuticals as a sterile, single-dose, lyophilized powder for prolonged-release injectable suspension (Abilify Maintena^®^) or a ready-to-use aqueous suspension (Abilify Asimtufii^®^) for the treatment of schizophrenia in adults. AM possesses robust thermal stability and predictable dehydration behavior. It undergoes a controlled dehydration process, transitioning to an anhydrous form only at elevated temperatures (ca. 124 °C), preserving its crystalline integrity during bead milling [37]. Moreover, unlike its anhydrous or amorphous counterparts, AM is less prone to polymorphic transitions under manufacturing or storage conditions, ensuring a predictable pharmacokinetic profile [38,39,40].

## 2. Materials and Methods

### 2.1. Materials

Aripiprazole monohydrate was manufactured in-house and obtained in bulk form, with varying initial particle sizes. Sodium carboxymethylcellulose sodium salt (Aqualon CMC 7L2P) was sourced from Ashland (Hopewell, VA, USA). Mannitol and disodium phosphate monohydrate were sourced from Merck (Darmstadt, Germany). Water for injection (WFI) was used for all aqueous preparations. All materials were of pharmaceutical grade and used as received.

### 2.2. Batch Preparation Processes

The composition of the suspension was based on the composition of Abilify Maintena by Otsuka Pharmaceutical Netherlands B.V. (Amsterdam, The Netherlands) [41].

#### 2.2.1. Preparation of Vehicle

Carboxymethyl cellulose sodium salt (13.09 g) was slowly added to WFI (1300 g) and stirred for 1 h to complete dissolution. Mannitol (65.46 g) and disodium phosphate monohydrate (1.17 g) were then added, and the mixture was stirred for 10 min. The resulting solution was made up with water for injection to a total mass of 2770 g, and pH was adjusted from 6.2 to 7 (10% NaOH_aq_, 3 mL). Afterwards, the solution was filtered through a 0.22 µm filter (steam-sterilized KA02ECVP8G filter capsule from Pall (Fribourg, Switzerland)) and collected into a 5 L glass bottle.

#### 2.2.2. Vehicle Autoclaving

In the process of the preparation of the AM suspension, vehicle solution was autoclaved in laboratory autoclave 5075ELV from Tuttnauer Europe B.V. (Breda, The Netherlands) in a glass bottle at 121 °C for 15 min and cooled to ambient temperature. The preparation of vehicle solution for CMCNa molecular mass determination involved autoclaving for 15–60 min at 121 °C followed by cooling to ambient temperature.

#### 2.2.3. Preparation of Primary Suspension

Vehicle solution was transferred to high-shear rotor–stator homogenizer KappaVita HM5 from Netzsch (Weyhe-Dreye, Germany). Aripiprazole monohydrate (327.24 g) was then introduced into the vehicle solution and homogenized at 12,000 rpm (31 m/s) for 40–60 min. The temperature during homogenization was maintained at 30 °C using a cooling jacket. The particle size of aripiprazole monohydrate was monitored offline on a sample collected from the homogenizing tank every 20 min. The process was finished when D[4;3] of approximately 12 µm was achieved (Dv100 below 100 µm).

#### 2.2.4. Preparation of Secondary Suspension

Homogenizing tank KappaVita HM5 was connected through silicone hoses with bead mill LabStar LS1 from Netzsch (Weyhe-Dreye, Germany) equipped with a milling chamber (120 mL) filled yttrium-stabilized zirconium oxide milling beads (0.5 mm) from Netzsch. The milling bead load was 45% of the nominal milling chamber volume, and the primary suspension was circulated between the two units using a peristaltic pump. Wet milling was performed at a mill tip speed of 1000 rpm, suspension flow rate of 140 g/min and homogenizer rotor speed of 10,000 rpm until reaching a Dv50 value of about 3.30–3.50 µm. Suspension temperature was kept at 15 °C. Samples of the suspension were collected every 20 min for analysis to determine the milling endpoint based on the target Dv50 value. Towards the end of the milling process, more frequent sampling, i.e., every 10 min, may be required to achieve the desired particle size, like in the case of batch AMDP2 (see Table 1 for batch numbering). After achieving the desired particle size, a final high-shear homogenization step was conducted at 12,000 rpm for 10 min to achieve the final target PSD. The temperature of the suspension was kept below 40 °C by means of the water jacket cooling of the homogenizing tank. Each batch was divided into two sub-batches denoted with suffixes R and H. About half of the amount of the suspension was withdrawn to be filled in vials immediately after bead milling was stopped (suffix R). The other part of the suspension was subject to final high-shear homogenization for 10 min (suffix H).

#### 2.2.5. Freeze-Drying

Suspension in partially stoppered glass vials was freeze-dried in the Epsilon 2-6D LSCplus lyophilizer from Martin Christ (Osterode am Harz, Germany). The lyophilization program included the following stages: initial freezing to −35 °C for 1 h, followed by annealing at −18 °C and a primary drying phase starting at −35 °C and 0.25 mbar and reaching −10 °C. The total primary phase duration was 36 h. No secondary drying was necessary. Vials with lyophilizate were stoppered by lowering shelves.

#### 2.2.6. Sampling

Samples of each batch were analyzed for particle size distribution during all steps of suspension preparation, shortly after lyophilization and during the stability study. The viscosity of the reconstituted suspension was also monitored during the stability study.

#### 2.2.7. Batch Manufacturing Conditions and Naming

In order to evaluate the impact of thermal treatment and high-shear homogenization on particle comminution, four processes of the laboratory-scale manufacturing of the aripiprazole monohydrate drug product (AMDP) were conducted and analyzed (Table 1). Manufacturing processes were varied by the application of optional processes: AMDP-1 followed a conventional method without the autoclaving of a vehicle solution or high-shear homogenization of a suspension during bead milling, while AMDP-4 incorporated both processes. AMDP-2 and AMDP-3 utilized combinations of these conditions. All batches were manufactured using the same amounts of the aripiprazole monohydrate drug substance (AMDS) and excipients. Different batches of AMDS were used during the manufacture of the AMDPs, as listed in Table 1.

### 2.3. Analytical Methods

#### 2.3.1. Particle Size Distribution (PSD) Analysis

The particle size distribution of suspension samples was analyzed with laser diffraction using Mastersizer 3000 from Malvern Panalytical (Malvern, UK) equipped with a Hydro MV dispersing unit. A predispersion of about 0.5 mL of the analyzed suspension sample in 20 mL of WFI was added to the dispersing unit filled with WFI and sonicated for 20 s. Six measurement runs (10 s, Ri = 1.593) were performed in each analysis and averaged. The results were analyzed with a general-purpose analytical model for nonspherical particles (Mie theory) available in Mastersizer 3000 software.

The particle size distribution of aripiprazole monohydrate drug substance powder samples was analyzed using Mastersizer 2000 from Malvern Panalytical equipped with a Scirocco 2000 dispersing unit for dry powder samples. The powder sample (1 g) was placed in the standard sample tray equipped with a sieve without bearing balls. The slit width was 3 mm and the feed rate 60% at an air pressure of 1 bar; the measurement time was 6 s, and 3 measurement runs were performed in each analysis and averaged. The results were analyzed with a general-purpose model (Mie theory) available in Mastersizer 2000 software.

#### 2.3.2. Viscosity Analysis

Viscosity was measured using Brookfield-type viscometer DX3TLVKJO from Brookfield AMETEK (Middleborough, MA, USA). A total of 1.5 mL of the suspension sample was analyzed using the DIN-83 spindle and small sample adapter (7RP). Spindle rotation was increased from 50 to 250 rpm. Viscosity was calculated using the Bingham model available in Rheocalc T software version 1.2.19 for the data observed at 250 rpm at a sample temperature of 20.0 °C.

#### 2.3.3. CMCNa Molecular Mass Determination

Molecular mass analysis was performed by an SEC HPLC-MALS technique using the HPLC system from Waters (Milford, MA, USA), with Suprema Analytical 10000Å, with a 10 μm column 8 × 300 mm. Detectors used: Dawn 8+ Heleos II (MALS) and Optilab T-rEX (RI), both from Wyatt (Santa Barbara, CA, USA). Systems were run under the control of Empower 3.0 (Waters) and Astra 6.1.7.17 (Wyatt). The mobile phase used was composed of 17.5 mM Na_2_PO_4_ pH 7.0 and 0.05% sodium azide in water at a flow rate of 1 mL/min. Samples of 4.80 g of vehicle solution were lyophilized and dissolved in 10 mL of mobile phase, and 100 µL aliquots were analyzed.

#### 2.3.4. X-Ray Powder Diffraction (XRPD) Analysis

XRPD analysis was performed for powder AMDS and lyophilized AMDP. AMDP lyophilized cake was gently crushed with a pestle in an agate mortar before analysis with the X’Pert PRO diffractometer from Malvern Panalytical equipped with a CuKα 1.54 Å radiation source and nickel Kβ filter. Data was collected at a scan range of 4–40° 2Θ with a step size of 0.02°.

#### 2.3.5. Optical Microscopy

Microscopic images of diluted samples (50× dilution with WFI) were taken using Morphologi G2 from Malvern Panalytical at 50× magnification.

#### 2.3.6. Stability Study

Samples subject to the stability study were stored for 3 months in climatic chamber KBF 720 from Binder (Tuttlingen, Germany) at a temperature of 40 °C and relative humidity of 75% in glass vials closed with chlorobutyl rubber stoppers and protected with aluminum caps.

### 2.4. Statistical Analysis

All experimental data for processes AMDP-1, AMDP-2 and AMDP-4 is presented as means (*n* = 3) ± standard error. Data for the process AMDP-3 and stability data are presented for single batches. The differentiation of results was performed by one-way ANOVA analysis (α = 0.05) followed by a post hoc Tukey’s HSD test for pairwise comparisons (α = 0.05). Results for AMDP-3 were compared with results for other processes using a one-sample Student’s *t*-test (α = 0.05).

## 3. Results

The AMDS batches used for the manufacture of AMDP differed only by particle size distribution, as shown in Table 2.

An SEC-MALS study of CMCNa molecular weight was performed to assess the impact of the heating of its solution to 121 °C. It was found that although the heating process has a limited effect on all parameters describing molecular mass distribution (Mn, Mw, Mz), no significant change is observed in heating over time (Table 3). Similarly, the polydispersity index did not show any significant change.

Before each milling process, pre-milling homogenization of AM with the vehicle solution was performed. The main objective of this step is the formation of a primary, uniform suspension of AM in the vehicle solution. The suspension at this point contains a wide distribution of particle sizes, largely dependent on the crystal sizes of the AMDS batch. If some particles are larger than the pores of the sieve installed in the bead milling chamber, it leads to the clogging of the milling chamber, a rapid pressure increase above safety limits and the inability to continue the milling process. It may thus be necessary to reduce the particle size of the AMDS before the start of the milling process. The safest approach is to assure that no particle is larger than the sieve pores’ diameter. In practice, in order to meet this requirement, the milling process was started only when Dv100 was below 100 µm (diameter of the sieve installed in the milling chamber). An additional benefit from this strategy is the normalization of particle size distribution at the beginning of bead milling across AMDS batches with different PSDs and thus the unification of the milling times required to process different AMDS batches to reach the required PSD. The results for PSD measurements at the end of the pre-milling homogenization step are presented in Table 4. It should be noted that this step yielded generally non-significant differences in mean PSD parameters between processes (*p* > 0.05). The results for AMDP-3 do not differ significantly from other processes except for Dv99, for which the result differs from AMDP-1 and AMDP-2 (*p* < 0.05).

The PSD of the particles in suspension was measured at several timepoints during the milling process. In order to be able to compare different milling processes, theoretical passages of the suspension were calculated as follows:

(1) $$N_{n}=\frac{\dot{m}t_{n}}{m_{susp,n}}$$

where

*t_n_*—total time of milling for the *n*-th sample [min];
*ṁ*—mass flow rate [g/min];
*m_susp,n_*—mass of the remaining suspension at the *n*-th sample, accounting for the total mass of previous samples [g].

**Figure 1:**
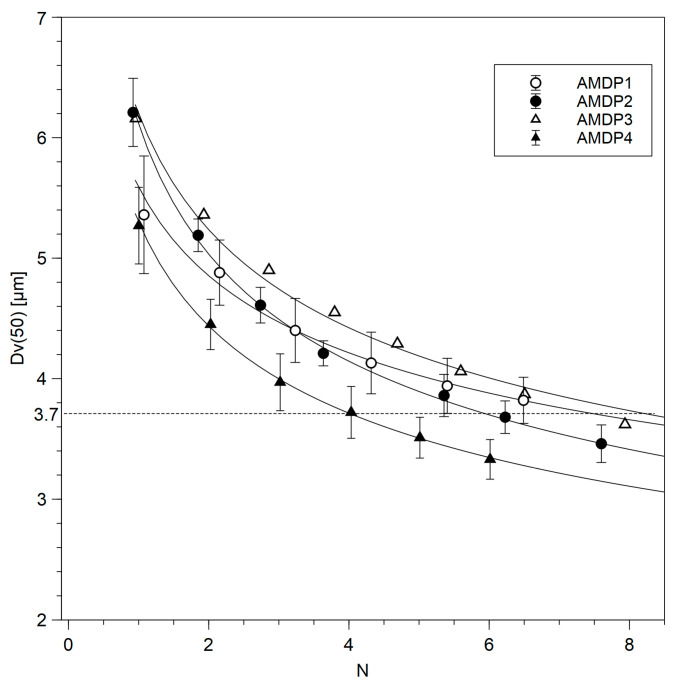
Particle size decrease (Dv50) as a function of theoretical passages (*N*) during the milling process. Dv50 = 3.7 µm is marked with dashed line.

The observed particle size decrease, as Dv50 as a function of theoretical passages *N*, is presented in Figure 1. Data for each batch was fitted with the following exponential function [42]:

Dv50 = *a^N^* + *b*, (2)

where

*N*—theoretical passages;
*a*, *b*—fitting parameters.

A comparison of the PSD of differently treated suspensions measured before (pre-lyo) and after lyophilization (post-lyo) as well as the viscosity of the reconstituted suspension after lyophilization is given in Table 5. As can be expected, the highest decrease in particle size during the post-milling high-shear homogenization step is observed for batches for which such a process was not employed during bead milling, i.e., AMDP-1 (*p* < 0.05) and AMDP-3 (not evaluated statistically). Differences observed for AMDP-2 and AMDP-4 are not significant (*p* > 0.05). The viscosity of reconstituted samples is also observed to be lower for H variants of AMDP-1 (*p* < 0.05 for comparison with AMDP-2 and AMDP-4) and AMDP-3 (not evaluated statistically). The PSDs of the batches manufactured in each of the processes, AMDP-1 to AMDP-4, do not differ significantly due to the fact that the process was, in each case, terminated after achieving the target Dv50. What, however, distinguishes the analyzed processes is the number of passages of the suspension through the milling chamber required to achieve a certain Dv50. Since the target PSD was not achieved in all our processes during bead milling, we have chosen Dv50 = 3.7 µm as the value for which the number of required passages is compared as a measure of process rate. This estimation is shown in Table 6.

Lyophilized samples of one batch of each process were subjected to a stability study as described in Section 2. The results are presented in Table 7. For PSD, only the D[4;3] parameter is presented as a measure of overall size with emphasis on the fraction of larger particles. A significant increase in viscosity of more than 5 mPa·s is observed for both variants of AMDP-1 and a much smaller increase, ca. 2 mPa·s, for AMDP-3R. For other batches, the viscosity change is about 1 mPa·s or less and considered negligible. Particle size, expressed as D[4;3], is observed to increase most significantly for batches AMDP-1 in both variants, for AMDP-2H and, to a lesser extent, for AMDP-3R.

**Figure 2:**
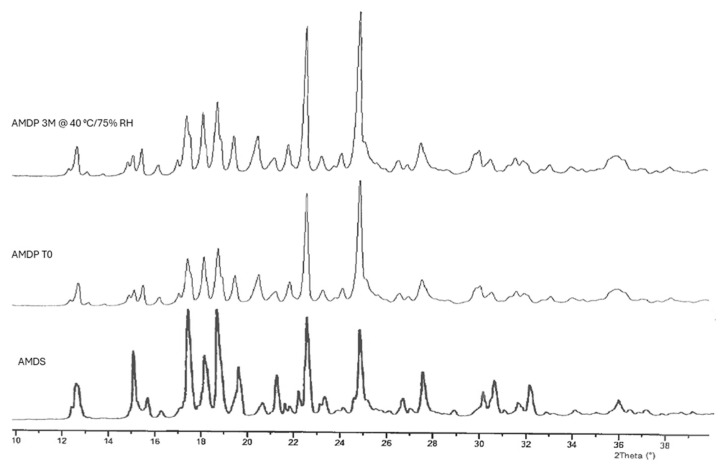
Exemplary X-ray powder diffractograms of AM in powder form, before formulation process (AMDS), AM in drug product after lyophilization, before stability study (AMDP T0), and AM in drug product after 3 months of stability study at 40 °C/75% RH (AMDP 3M @ 40 °C/75% RH).

An exemplary XRPD study result presented in Figure 2 for a batch manufactured according to the conditions of AMPD-4H indicates the stability of the monohydrate form during all steps of manufacture, as well as during prolonged storage at a temperature as high as 40 °C.

## 4. Discussion

**Figure 3:**
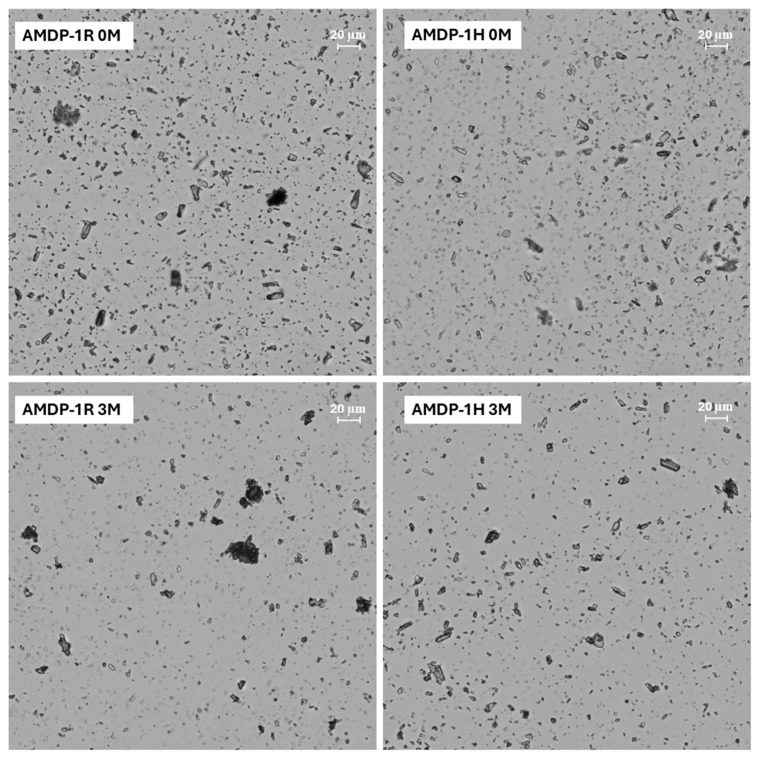
Microscopic images of particles obtained from AMDP-1 process (50× magnification); AMDP-1R 0M: after bead milling, without post-milling homogenization; AMDP-1H 0M: after post-milling homogenization; AMDP-1R 3M: after bead milling, without post-milling homogenization after 3 months at 40 °C/75% RH; AMDP-1H 3M: after post-milling homogenization after 3 months at 40 °C/75% RH.

The experimental results presented above show several benefits of introducing the supporting processes, i.e., thermal treatment of the vehicle solution and high-shear homogenization, into AMDP manufacture. Starting at the vehicle preparation stage, the introduction of thermal treatment creates the possibility of obtaining a sterile solution via the most reliable method, i.e., thermal sterilization, if one decides to follow the required sterility assurance procedures. Notably, the vehicle solution sterilization step, be it autoclaving or sterilization by filtration, is seldom employed in laboratory processes reported in the scientific literature, even if subsequent absorption and/or irritation studies are conducted. It needs, however, to be stressed that following AMDS addition to the vehicle solution, in order to maintain the sterile status of the preparation, the process needs to be executed in aseptic conditions. No sterility assurance procedures were necessary in this work, and thus they were not followed; however, it is shown that the thermal sterilization of a CMCNa-containing solution is a viable option. Importantly, it is shown that CMCNa is only slightly affected by the heat treatment. Usually, a decrease in the viscosity of CMCNa-containing solutions is observed during storage, which is associated with the gradual hydrolysis of the cellulose chain. Such a process is much more pronounced at elevated temperatures [43,44]. The study performed in this work showed that only a minor change is observed in CMCNa molecular weight before and after a high-temperature cycle representing standard sterilization conditions i.e., heating to 121 °C for 15 min. What is important is that PDI did not change significantly upon heating, which is not characteristic of a typical hydrolysis process, in which one would expect Mw and Mz to decrease and Mn to increase over heating time as a result of the most probable hydrolysis of longest chains and the formation of shorter ones. No such observation could be made in this case, likely due to the fact that the CMCNa used possesses a low molecular weight and is used in a low concentration and at neutral pH, whereas a viscosity decrease causing the hydrolysis of cellulose chains is most pronounced for high-molecular-weight materials at acidic pH. Moreover, the results of the stability study performed in accelerated conditions, i.e., 40 °C/75% RH for 3 months, show stable viscosity results for the batches manufactured with the vehicle which underwent thermal treatment. The dynamic viscosity of a suspension is a result of a complex interplay of many effects, and a discussion of them is beyond the scope of this work [45]. A fact which is, however, worthy of note is that the observation of no significant changes in the viscosity of the suspension over time is a very clear sign that this subtle balance of forces keeping the reconstituted suspension stable for the time required for administration is maintained during storage when thermal treatment is employed, despite minor changes in the molecular weight of CMCNa. Notably, the viscosity of the reconstituted suspension for both AMDP-1R and, to a slightly lesser extent, AMDP-1H evidently increases over time, indicating a loss of stability in the suspension. This observation correlates with the increase in the D[4;3] parameter in the stability study, indicating the formation of larger particles for both process variants, with the R variant showing a more pronounced change. AMDP-1 was manufactured without thermal treatment of the vehicle solution and without high-shear homogenization during milling, which is a typical procedure found in the scientific literature describing studies on aripiprazole suspensions [39,46]. It is an established fact that during bead milling, the large forces exerted by beads on the particles in the suspension lead to their aggregation and that these aggregates can subsequently be crushed or left in the suspension, which is also confirmed by microscopic analysis performed for AMDP-1 (Figure 3) [47,48,49]. This effect is especially visible when one compares the PSD of suspensions of variants R and H before and after final homogenization. While the decrease in PSD parameters is observable after final homogenization for AMDP-1 and AMDP-3, where no homogenization was performed during bead milling, it is negligible for AMDP-2 and AMDP-4, where homogenization was employed. This observation correlates well with common knowledge about the aggregation process during bead milling, which needs to be reverted to assure the high quality and safety of the injectable preparation [47]. Allowing the formation of aggregates increases the risk of clogging in the needle during administration but also increases the amount of solid residue left in the vial after withdrawal, decreasing the amount of API administrable to the patient. The continuous disruption of aggregates forming during bead milling by high-shear homogenization also allows one to perform the milling process in a more repeatable manner, since lower amounts of secondary particles are impacted by the beads, leading to more predictable results. An obvious benefit of the employment of this process is the increase in the effectiveness of bead milling; e.g., when comparing the milling process of AMDP-1 and AMDP-2, the introduction of the homogenization of bead milling allowed for the continuation of an effective particle size decrease in AMDP-2 after 5.5 theoretical passages, when almost no change was observed for AMDP-1. High-shear homogenization in the equipment setup utilized in this work is not capable of an effective size decrease in particles below the range shown in Table 4; thus, its positive effect can only be attributed to the disruption of secondary particles. Generally, the use of any of the two processes—vehicle heat treatment and high-shear homogenization during milling—shows a decrease in secondary particle formation, which is manifested in the reduction in the PSD change in the R and H variants of AMDP-2 and AMDP-3 compared to AMDP-1. The very good stability of both PSD and viscosity in AMDP-4 can be viewed as a manifestation of the synergy of these effects. A comparison of the number of theoretical passages required to achieve an arbitrarily selected value of Dv50 = 3.7 µm shows that the conditions of the AMDP-1 process allow for a significant reduction in the bead milling time and, as a result, may possibly also lower the energy cost of the process. Finally, it is also shown that, similarly to in earlier reports, AM crystal structure is not altered during bead milling [39], high-shear homogenization and lyophilization in the worst-case conditions used in the present work, i.e., those used for batch AMDP-4H, making the discussed process not only effective but also safe from the point of view of the polymorphic purity of AM.

## 5. Conclusions

In conclusion, we have shown the importance of careful evaluation of laboratory-scale manufacturing processes of pharmaceutical suspensions in order to ensure that the resulting products can be safely stored until further studies. Secondly, the feasibility and safety of the most reliable sterilization technique, i.e., the moist heat sterilization of CMCNa-containing vehicle solutions, was confirmed. As a result, we have developed a robust and effective manufacturing process for the model drug product containing aripiprazole monohydrate and, through the use of additional processes—vehicle thermal treatment and high-shear homogenization during bead milling—ensured its stability. The main benefit of this research is that both processes are easy to scale and commonly used in the pharmaceutical industry. We believe the proposed solutions can be seamlessly applied to the production of LAI suspensions.

## 6. Patents

The work reported herein is subject to patent application “Method of preparation of a pharmaceutical composition comprising aripiprazole”, EP4291166 A1.

## Figures and Tables

**Table 1:** Overview of AMDP batches, showing the application of optional processes.

AMDS Batch Name ^1^	AMDP Batch Name ^2^	Vehicle Thermal Treatment	Homogenization During Bead Milling	Homogenization After Bead Milling
AMDS-1	AMDP-1R	N	N	N
	AMDP-1H			Y
AMDS-2	AMDP-2R	N	Y	N
	AMDP-2H			Y
AMDS-2	AMDP-3R	Y	N	N
	AMDP-3H			Y
AMDS-3	AMDP-4R	Y	Y	N
	AMDP-4H			Y

**^1^** AMDS: aripiprazole monohydrate drug substance used to manufacture drug product; **^2^** AMDP: aripiprazole monohydrate drug product.

**Table 2:** Particle size distribution of different AMDS batches used in the manufacture of AMDPs.

AMDS Batch Name	Dv10 [µm]	Dv50 [µm]	Dv90 [µm]
AMDS-1	24	166	467
AMDS-2	22	192	548
AMDS-3	9	55	215

**Table 3:** Molecular weight distribution parameters (Mn, Mw, Mz) and polydispersity index of CMCNa solution before and after thermal treatment at 121 °C.

Thermal Treatment Duration [min]	Mn [kDa]	Mw [kDa]	Mz [kDa]	PDI (Mw/Mn)	PDI (Mz/Mn)
0	40.5 (± 1.6)	60.8 (±3.5)	96.6 (±10.6)	1.50 (±0.06)	2.39 (±0.23)
15	41.1 (±2.8)	62.3 (±3.0)	103.7 (±6.3)	1.52 (±0.03)	2.52 (±0.07)
22	39.1 (±1.8)	60.4 (±1.3)	99.6 (±2.7)	1.54 (±0.05)	2.55 (±0.14)
30	39.6 (±1.2)	60.5 (±2.8)	99.9 (±10.3)	1.53 (±0.04)	2.52 (±0.20)
60	40.1 (±1.9)	59.4 (±1.5)	95.5 (±3.6)	1.48 (±0.04)	2.39 (±0.13)

**Table 4:** Particle size distribution at the end of the pre-milling homogenization step. Mean values (±SE) are given (*n* = 3). For AMDP-3, *n* = 1.

Manufacturing Process	Dv10 [µm]	Dv50 [µm]	Dv90 [µm]	Dv99 [µm]	Dv100 [µm]	D[4;3] [µm]
AMDP-1	1.75 (±0.04)	7.31 (±0.25)	29.9 (±0.8)	54.7 (±0.2)	80.8 (±2.9)	12.1 (±0.3)
AMDP-2	1.74 (±0.07)	8.51 (±0.35)	34.5 (±1.1)	62.7 (±1.1)	92.6 (±3.3)	14.0 (±0.5)
AMDP-3	1.86	7.49	30.3	57.1	85.6	12.4
AMDP-4	1.60 (±0.07)	7.77 (±0.76)	31.8 (±1.9)	56.4 (±2.5)	81.8 (±3.1)	12.9 (±0.9)

**Table 5:** Comparison of particle size distribution before (pre-lyo) and after (post-lyo) lyophilization and viscosity of the reconstituted suspension. Mean values (±SE) are given (*n* = 3). For AMDP-3, *n* = 1.

Process	Stage	Dv10 [µm]		Dv50 [µm]		Dv90 [µm]		D[4;3] [µm]		Viscosity [mPa·s]	
		R ^1^	H ^1^	R ^1^	H ^1^	R ^1^	H ^1^	R ^1^	H ^1^	R ^1^	H ^1^
AMDP-1	pre-lyo	1.30 (±0.09)	1.21 (±0.07)	3.81 (±0.12)	3.36 (±0.08)	10.05 (±0.49)	8.37 (±0.31)	4.93 (±0.21)	4.30 (±0.08)	- ^3^	- ^3^
	post-lyo	1.25 (±0.08)	1.20 (±0.06)	3.50 (±0.10)	3.30 (±0.07)	9.36 (±0.40)	8.25 (±0.28)	4.61 (±0.17)	4.27 (±0.06)	14.5 (±0.2)	13.3 (±0.3)
AMDP-2	pre-lyo	1.31 (±0.01)	1.29 (±0.02)	3.46 (±0.09)	3.51 (±0.11)	9.27 (±0.38)	9.40 (±0.53)	4.65 (±0.13)	4.73 (±0.20)	- ^3^	- ^3^
	post-lyo	- ^2^	1.30 (±0.02)	- ^2^	3.41 (±0.11)	- ^2^	9.34 (±0.61)	- ^2^	4.69 (±0.21)	- ^2,3^	12.5 (±0.4)
AMDP-3	pre-lyo	1.46	1.37	3.62	3.39	9.06	8.58	4.63	4.49	- ^3^	- ^3^
	post-lyo	1.40	1.35	3.42	3.31	8.81	8.42	4.53	4.42	11.7	11.1
AMDP-4	pre-lyo	1.18 (±0.03)	1.16 (±0.05)	3.33 (±0.10)	3.36 (±0.11)	9.19 (±0.44)	9.46 (±0.46)	4.73 (±0.18)	4.76 (±0.13)	- ^3^	- ^3^
	post-lyo	- ^2^	1.12 (±0.07)	- ^2^	3.21 (±0.15)	- ^2^	9.16 (±0.79)	- ^2^	4.49 (±0.31)	- ^2,3^	11.2 (±0.3)

^1^ R: batch variant without homogenization after bead milling; H: batch variant with homogenization after bead milling. ^2^ R: batch variant for which the batch was not lyophilized. ^3^ Comparison of viscosity of suspension before filling into vials and reconstituted suspension after lyophilization is not informative due to significant difference in concentration.

**Table 6:** Number of theoretical passages of suspension through the milling chamber (*N*) required to reach Dv50 = 3.7 µm.

Manufacturing Process	*N*
AMDP-1	8
AMDP-2	6
AMDP-3	7
AMDP-4	4

**Table 7:** Stability study results: D[4;3] and viscosity over 3 months at 40 °C and 75% relative humidity.

Manufacturing Process	Timepoint [months]	D[4;3] [µm]		Viscosity [mPa·s]	
		R	H	R	H
AMDP-1	0	4.67	4.19	14.9	13.2
	3	5.09	4.45	21.7	18.6
AMDP-2	0	- ^1^	4.33	- ^1^	11.7
	3	- ^1^	4.85	- ^1^	12.1
AMDP-3	0	4.53	4.42	11.7	11.1
	3	4.75	4.43	13.7	12.3
AMDP-4	0	- ^1^	3.89	- ^1^	10.7
	3	- ^1^	3.79	- ^1^	11.3

^1^ R: batch variant (without final high-shear homogenization) was not lyophilized.

## Data Availability

The data that support the findings of this study are available from the corresponding author, upon reasonable request.
